# NutriRisKids-ICU: Development and content validation of a nutritional risk screening tool for critically ill children and adolescents

**DOI:** 10.1590/1984-0462/2026/44/2025246

**Published:** 2026-04-03

**Authors:** Verônica Ramos Souza, Vanessa Camargo Trida, Beatriz Polisel Mazzoni, Adriana Servilha Gandolfo, Werther Brunow de Carvalho, Artur Figueiredo Delgado, Patrícia Zamberlan

**Affiliations:** a Universidade de São Paulo – São Paulo, SP, Brazil.

**Keywords:** Pediatric intensive care units, Malnutrition, Delphi technique, Screening, Nutritional assessment, Child nutrition, Unidades de terapia intensiva pediátrica, Desnutrição, Técnica Delphi, Triagem, Avaliação nutricional, Nutrição da criança

## Abstract

**Objective:**

The aim of this study was to develop and validate the content of a nutritional risk screening tool for critically ill children and adolescents in pediatric intensive care units (PICUs).

**Methods:**

Care-convergent study for the development and content validation of an instrument for assessing nutritional risk in children admitted to the PICU. The initial development was based on a literature review and the clinical experience of the authors. Content validation was conducted using the Delphi technique in two successive rounds with intentionally selected experts, each with a minimum of 5 years of experience in pediatric intensive nutritional therapy (NT). Consensus analysis was performed using the content validity index (CVI), with minimum values of 0.70 for individual items and 0.90 for the overall instrument.

**Results:**

Validation occurred in two rounds. In the first round, 18 experts suggested adjustments, and the revised version was reassessed by 12 experts in the second round. The final tool achieved an overall CVI of 0.958. The validated version included age subgroups, presence of chronic diseases, length of prior hospitalization, subjective impression of malnutrition risk, type of NT at admission, and C-reactive protein (CRP) levels.

**Conclusions:**

The nutritional risk screening tool for critically ill pediatric patients, named *NutriRisKids-ICU*, was developed, and its content was validated by expert consensus. The tool combines theoretical evidence and clinical expertise, showing potential for application in PICUs. However, further studies are needed to assess its applicability, sensitivity, and specificity.

## RESUMO

**Objetivo:**

Desenvolver e validar o conteúdo de um instrumento de triagem de risco nutricional para crianças e adolescentes gravemente doentes em Unidades de Terapia Intensiva Pediátrica (UTIP).

**Métodos:**

Estudo do tipo convergente assistencial de desenvolvimento e validação de conteúdo de instrumento para avaliação do risco nutricional em crianças internadas em UTIP. A elaboração inicial baseou-se em revisão de literatura e na experiência clínica dos autores. A validação de conteúdo foi conduzida por meio da técnica Delphi, em duas rodadas sucessivas, com especialistas selecionados intencionalmente, considerando experiência mínima de cinco anos em Terapia Nutricional (TN) pediátrica intensiva. A análise de consenso foi realizada pelo Índice de Validade de Conteúdo (CVI), adotando-se valores mínimos de 0,70 por item e 0,90 para o instrumento global.

**Resultados:**

A validação ocorreu em duas rodadas. Na 1ª, 18 especialistas propuseram ajustes, sendo a versão revisada reavaliada por 12 especialistas na 2ª rodada. O instrumento final apresentou IVC geral de 0,958. A versão final incluiu subgrupos etários, presença de doenças crônicas, tempo de hospitalização prévia, impressão subjetiva de risco de desnutrição, tipo de TN na admissão e níveis de proteína C-reativa (PCR).

**Conclusões:**

O instrumento de triagem nutricional para pacientes críticos pediátricos, denominado NutriRisKids-ICU, foi desenvolvido e teve seu conteúdo validado por consenso de especialistas. A ferramenta combina evidências teóricas e experiência clínica, apresentando potencial de uso em UTIP. Entretanto, estudos adicionais são necessários para avaliar sua aplicabilidade, sensibilidade e especificidade.

## INTRODUCTION

 Critically ill patients admitted to intensive care units (ICUs) often present with vital dysfunctions — renal, neurological, cardiovascular, metabolic, and/or respiratory—that result in clinical instability and require invasive procedures, increasing fragility and the risk of additional complications.^1,2^ In pediatrics, these impacts may extend beyond hospitalization, compromising development throughout life.^3^

 The metabolic stress resulting from critical illness is characterized by increased energy metabolism and marked protein catabolism, followed by a stabilization phase, still with catabolism present, and later by a recovery phase, marked by the onset of protein anabolism.^4^ Nutritional status plays a decisive role in this process, with malnutrition being associated with prolonged mechanical ventilation, longer hospital stays, increased risk of infections, higher mortality, and elevated hospital costs.^5-7^

 Studies indicate that more than half of pediatric patients present nutritional deficits already at admission to the pediatric intensive care unit (PICU), and that this risk is often not identified by the healthcare team.^8^ In this context, nutritional screening assumes a strategic role, as it enables the early detection of children and adolescents at risk of nutritional deterioration from admission, favoring the timely initiation of nutritional therapy (NT). This approach is essential to prevent or minimize clinical complications such as prolonged mechanical ventilation, infections, and increased mortality.^9^

 The most frequently cited pediatric nutritional screening tools in the literature include the Nutritional Risk Score (NRS), the Pediatric Nutritional Risk Score (PNRS), the Pediatric Yorkhill Malnutrition Score (PYMS), the Screening Tool for Risk on Nutritional Status and Growth (STRONGkids), and the Screening Tool for the Assessment of Malnutrition in Pediatrics (STAMP).^10^ These tools assess factors such as disease severity, weight loss, gastrointestinal alterations, and visible signs of nutritional depletion. However, none of them were specifically developed or validated for critically ill children and adolescents in the PICU, where metabolic stress, inflammation, and therapeutic interruptions strongly influence nutritional outcomes.^11^

 Despite its relevance, there is a gap in the availability of validated and specific screening instruments for critically ill pediatric patients, who present clinical, metabolic, and nutritional particularities distinct from those of other hospitalized children. This scenario reinforces the urgent need for tools adapted to the reality of PICUs, capable of ensuring precise risk identification and the implementation of timely and targeted nutritional interventions.^12^

 Therefore, the present study aims to develop and validate a nutritional screening tool specific to critically ill children and adolescents admitted to PICUs, in order to improve early identification of nutritional risk and support interventions that contribute to better clinical outcomes. 

## METHOD

 This is a care-convergence–type study in which the chosen validation technique was the Delphi method, widely used for the development and validation of assessment instruments.^13,14^ One of its advantages is the possibility of gathering opinions from experts with different regional backgrounds and experiences.^15^

 Participants were selected through intentional (non-probabilistic) sampling, considering verified professional experience in pediatric intensive NT (minimum of 5 years) and involvement in clinical practice, teaching, or research in the field. This strategy aimed to ensure that the panel consisted of experts with theoretical and practical mastery of the content being evaluated. 

 The number of participating experts (n=18 in the first round and n=12 in the second) was determined according to methodological recommendations for content validation studies using the Delphi technique, which suggest panels of 10–30 experts, depending on the complexity of the topic and the heterogeneity of the group.^16,17^

 Initial recruitment was conducted among members of the Brazilian Intensive Care Medicine Association-Pediatrics, using the snowball sampling technique.^18^ To minimize potential biases inherent to this method, efforts were made to diversify participants in terms of geographic region, institutional affiliation, and professional area (nutrition, medicine, nursing, and physical therapy). In addition, responses were collected anonymously to ensure independence of opinion and prevent influence among evaluators. This strategy allowed for the formation of a representative and multidisciplinary panel of experts, supporting a robust technical consensus for the content validation of the instrument. 

 The initial development phase consisted of a literature review in scientific databases, including Scientific Electronic Library Online (SciELO), PubMed-National Institutes of Health (NIH), Google Scholar, and the Virtual Health Library (BVS). This review identified clinical, nutritional, and metabolic changes occurring in critically ill children that are associated with clinical outcomes. Based on these findings, an initial version of the instrument was created and refined before being sent to experts for evaluation. 

 The instrument was structured into six main domains, defined based on the literature and expert consensus, each representing a key determinant of nutritional risk in critically ill pediatric patients:

- Age: reflecting metabolic vulnerability and differing energy requirements across specific age groups;
- Chronic disease: which influences inflammatory status, metabolism, and protein-energy balance;
- Length of prior hospitalization: serving as a marker of cumulative catabolism and risk of nutritional depletion;
- Subjective impression of malnutrition: based on overall clinical assessment and observation of nutritional status;
- Type of NT: considering route, adequacy, and interruptions in nutrient provision;
- Inflammatory marker: reflecting the metabolic response to stress and the risk of nutritional deterioration.

Each domain consisted of items graded according to their clinical relevance and frequency observed in pediatric intensive care practice. Thus, the initial screening was as follows:

Item 1: Age: ≤2 years or >2 years;
Item 2: Presence of underlying disease: Yes or No;
Item 3: Days of hospitalization prior to PICU admission: ≥1 day or <1 day;
Item 4: Subjective impression of nutritional status (the patient appears to have a nutritional deficit): Yes or No;
Item 5: NT at the time of screening: Yes or No;
Item 6: CRP level at PICU admission: ≥50 mg/L or <50 mg/L.
Items 1–6 had two response options; each was assigned a specific score, whose sum determined nutritional risk classification as low, medium, or high.
Item 7: in turn, consisted of nutritional management strategies according to this classification: high risk — initiation of NT [enteral nutritional therapy (ENT) or parenteral nutritional therapy (PNT)]; medium risk — initiation of ENT; low risk—maintenance of regular diet with periodic reassessment.

Data collection was carried out by sending the screening instrument to experts, accompanied by a multiple-choice questionnaire developed in Google Forms®. Considering the appropriateness for small samples and the short response time, an adapted 3-point Likert scale was used. The questionnaire was distributed to 30 experts, anticipating possible losses during the process.^13,19^

 The questionnaire included a specific space for experts to suggest improvements for each item of the instrument. Experts were asked to evaluate two main aspects of each item: Relevance — whether the item was essential to achieve the proposed objective, and Clarity — whether the item was easily understandable and applicable.^20^

 Responses were recorded on a 3-point Likert scale, as follows: no = 1 point; maybe = 2 points; yes = 3 points. These responses were converted into numerical values for calculating the content validity index (CVI), which quantifies the level of agreement among evaluators.^19^ In this process, each "yes" response (indicating that the item was relevant or adequate) was coded as 1, and "no" and "maybe" responses (indicating disagreement) were coded as 0. 

 The validation process was based on expert consensus, determined through statistical convergence of CVI values. Notably, two types of CVI were used: the Item-level CVI (I-CVI), which is the proportion of agreement among participants for each item, obtained by the mean of binary scores (1 = yes, 0 = no/maybe), and the Total CVI (T-CVI), which is the overall agreement for the entire instrument, calculated as the mean of I-CVI values of all items.^14^ The standard deviation (SD) was also calculated to assess the variability of responses. A low SD indicated strong consensus among evaluators, while a high SD suggested greater divergence, even if the mean met the established cutoff point. Finally, the overall performance of the tool was assessed through the mean values of T-CVI for relevance and clarity. 

 There is no universally accepted cutoff point for CVI in the literature. However, consensus is considered to exist when less than one-third of evaluators disagree on a given item.^13^ In this study, the following parameters were adopted: minimum I-CVI per item: 0.70 (70% agreement) and minimum T-CVI: 0.90 (90% agreement) 

**Figure 1:**
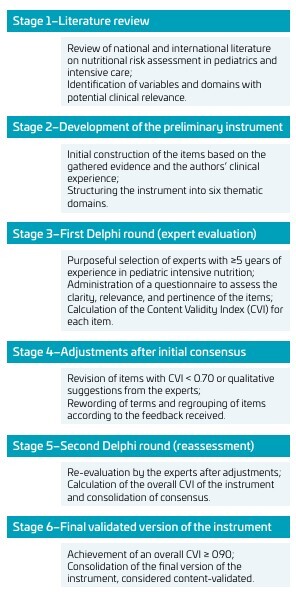
Steps in the process of development and content validation of the instrument for assessing nutritional risk in children admitted to a Pediatric Intensive Unit Care.

Figure 1 illustrates the main stages in the development and content validation of the instrument for assessing nutritional risk in children admitted to a PICU. The flowchart outlines the process from the initial conception and literature review, through item construction, expert evaluation, and the consolidation of the final validated version, providing a clear overview of the methodological steps undertaken. 

 This study was conducted in accordance with the ethical principles and guidelines for research involving human subjects. The project was submitted to the Research Ethics Committee (CAPPesq) of the institution and approved under CAAE 78777624.3.0000.0068. 

 To ensure confidentiality and voluntary participation, all experts involved in the validation process received the informed consent form (ICF) together with the screening instrument and the questionnaire. Participants provided explicit consent before responding, and their identities were kept confidential throughout the study. 

## RESULTS

 Invitations and questionnaires were sent to 30 experts, yielding a 60% response rate (18 participants) in the first round. Although this number may seem modest, a meta-analysis of educational studies found that research conducted exclusively online yields an average response rate of 44.1%. This result may be related to the more specific definition of the target population, a factor highlighted in the literature as capable of increasing adherence to questionnaires.^11^ Respondents included 11 physicians, 5 nutritionists, 1 physical therapist, and 1 nurse, representing three states in the country. 

 The T-CVI was calculated considering the criteria of clarity and relevance. Results from the first round showed a T-CVI of 83.3%, obtained from the mean values of clarity (79.4%) and relevance (87.3%). These results are presented in Tables 1 and 2. Comments and suggestions by item included:

- Item 1 (age): observations regarding clarity and suggestions to group distinct age ranges;
- Item 2 (preexisting clinical condition): recommendations to specify whether only the underlying disease or its exacerbation should be considered;
- Item 3 (days of hospitalization before PICU): suggestion to increase the number of days considered, since the longer the hospitalization, the higher the nutritional risk;
- Item 4 (subjective assessment of nutritional status): criticism regarding the subjectivity of visual evaluation, suggesting the inclusion of anthropometric measurements;
- Item 5 (current NT): recommendation to consider the need for supplements, modules, or hypercaloric formulas/diets;
- Item 6 (CRP at admission): suggestion to analyze other laboratory tests, such as lactate, procalcitonin, and ferritin;
- Item 7 (nutritional management strategies): comments on scoring and suggestions for adjustments in intervention strategies.

**Table 1:** Content Validity Index data by item and total for the concepts of relevance in the first and second rounds with the experts.

Concept	I-CVI relevance			
	Item 1	Item 2	Item 3	Item 4
1st round	0.72 (±SD 0.45)	0.89 (±SD 0.31)	0.94 (±SD 0.23)	1.00 (±SD 0)
2nd round	0.92 (±SD 0.28)	1.00 (±SD 0)	1.00 (±SD 0)	1.00 (±SD 0)
	Item 5	Item 6	Item 7	T- CVI (%)
1st round	0.94 (±SD 0.23)	0.72 (±SD 0.45)	0.89 (±SD 0.31)	87.3 (±SD 17.1)
2nd round	0.92 (±SD 0.28)	0.75 (±SD 0.43)	1.00 (±SD 0)	94.0 (±SD 9.14)

Item 1: age; Item 2: preexisting clinical condition; Item 3: days of hospitalization before PICU; Item 4: subjective assessment of nutritional status; Item 5: current NT; Item 6: CRP at admission; Item 7: nutritional management strategies.

I-CVI: Item-level Content Validity Index; SD: standard deviation; T-CVI: Total Content Validity Index.

**Table 2:** Content Validity Index data by item and total for the concepts of clarity in the first and second rounds with the experts.

Concept	I-CVI clarity			
	Item 1	Item 2	Item 3	Item 4
1st round	0.61 (±SD 0.49)	0.94 (±SD 0.23)	0.72 (±SD 0.45)	0.88 (±SD 0.31)
2nd round	1.00 (±SD 0)	1.00 (±SD 0)	1.00 (±SD 0)	0.92 (±SD 0.28)
	Item 5	Item 6	Item 7	T- CVI (%)
1st round	0.77 (±SD 0.42)	0.88 (±SD 0,31)	0.72 (±SD 0.45)	79.4 (±SD 20.9)
2nd round	0.92 (±SD 0.28)	1.00 (±SD 0)	1.00 (±SD 0)	97.6 (±SD 5.32)

Item 1: age; Item 2: preexisting clinical condition; Item 3: days of hospitalization before PICU; Item 4: subjective assessment of nutritional status; Item 5: current NT; Item 6: CRP at admission; Item 7: nutritional management strategies.

I-CVI: Item-level Content Validity Index; SD: standard deviation; T-CVI: Total Content Validity Index.

 After analyzing comments and CVI values, the tool was adjusted considering the experts’ suggestions, literature data, and clinical practice aspects, and then sent for a second round of evaluation. In this round, 12 experts who had participated in the first round were randomly selected: 7 physicians (2 professors and 5 clinicians — 4 with a PhD and 1 with a master’s degree), 3 nutritionists (all clinicians, 2 with a PhD), 1 physical therapist (clinician with a master’s degree), and 1 nurse (clinician pursuing a PhD). The median duration of experience in the PICU was 12 years (minimum = 5 years, maximum = 35 years). 

 In the second round, all items reached the minimum expected I-CVI of 0.70. The lowest relevance value was 0.75 in Item 6, but with no new suggestions. The overall CVI of the tool, obtained from the mean T-CVI values of clarity (97.6%) and relevance (94.0%), was 95.8%, validating the instrument according to the criteria established in the methodology (Tables 1 and 2). 

 To illustrate the applicability of the instrument, Table 3 was developed to present hypothetical examples of NutriRisKidsICU completion for three distinct patient profiles — low, moderate, and high nutritional risk. This example aims to demonstrate the scoring method and the resulting risk classification, highlighting the instrument’s potential to guide clinical decision-making from the moment of PICU admission. 

**Table 3:** Illustrative example of the application of the NutriRisKids-ICU instrument.

Patient profile	Clinical description	Total score	Risk classification	Recommended action
Patient A (low risk)	10 years old, no chronic disease, hospitalization <24 h, good nutritional status, oral diet, CRP 3 mg/L	2	Low	Maintain regular diet, reassess in 48 h
Patient B (moderate risk)	3 years old, stable congenital heart disease, 3 days of prior hospitalization, mildly underweight, use of EN via tube, CRP 8 mg/L	5	Moderate	Initiate/adjust ENT and monitor progression
Patient C (high risk)	6 months old, severe bronchiolitis, 5 days of prior hospitalization, malnourished, fasting, CRP 18 mg/L	8	High	Initiate early NT (ENT or PNT) and intensive nutritional monitoring

ENT: Enteral nutrition therapy; PNT: Parenteral nutrition therapy; CRP: C-reactive protein

**Figure 2:**
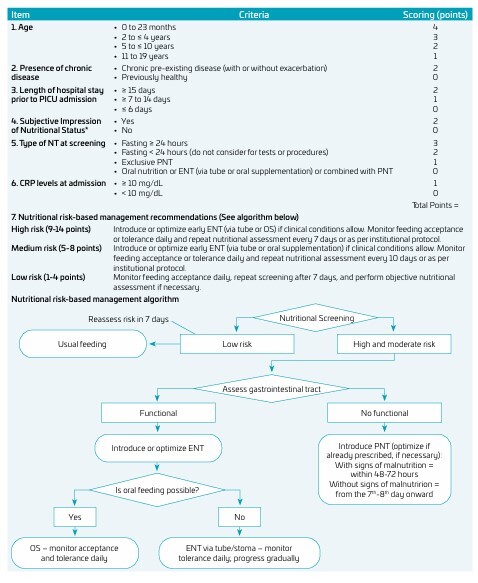
Nutritional risk screening for critically ill children and adolescents — NutriRisKids-ICU (final version). ENT: Enteral nutritional therapy; NT: Nutritional therapy; OS: Oral supplementation; PNT: Parenteral nutritional therapy. *Example: reduction of subcutaneous fat and/or muscle mass; emaciated face, prominent bones; dry skin; thin hair.

 After analysis and discussion, the experts’ suggestions were carefully incorporated into the instrument, resulting in the final version of the tool (Figure 2). 

## DISCUSSION

 The NutriRisKids-ICU, developed in this study, addresses this gap by integrating variables directly relevant to the metabolic and clinical complexity of pediatric critical illness. It includes six key variables — age, presence of chronic disease, duration of hospitalization prior to PICU admission, subjective assessment of nutritional status, type of NT, and CRP levels — identified through literature review and expert consensus. Unlike preexisting tools that rely heavily on anthropometric or non-routine biochemical parameters, NutriRisKids-ICU prioritizes practicality, rapid application, and feasibility in the early phase of PICU admission, when immediate decision-making is crucial.^21^

 The Delphi process enabled systematic refinement of each item, resulting in high agreement among experts. Adjustments were particularly significant for age- and disease-related variables, which initially presented lower clarity scores. The redefinition of age groups, based on physiological and developmental criteria, increased clarity and relevance of I-CVI values to above 90%, reflecting expert recognition that infants (28 days–2 years) are most vulnerable to malnutrition due to accelerated growth, limited reserves, and immature immunity,^22-24^ while adolescents are also at risk due to the nutritional demands of puberty.^25^

 Chronic disease was included as a distinct variable, acknowledging its impact on metabolic and nutritional status. The revised item, "preexisting chronic disease (with or without exacerbation)," aligns with evidence linking chronic conditions — such as congenital heart disease, cystic fibrosis, and chronic liver disease — to higher nutritional risk and poorer outcomes.^22^ Similarly, days of hospitalization prior to PICU admission were recognized as an indirect marker of cumulative catabolism, prolonged fasting, and infection risk, reinforcing their relevance in predicting nutritional deterioration. 

 The subjective assessment of nutritional status achieved strong expert consensus, highlighting the clinical value of experience-based evaluation in situations where anthropometry is not feasible, such as in edematous or ventilated patients. This aligns with prior evidence supporting clinical judgment as a valid and sensitive approach for early identification of malnutrition in critical care settings. 

 NT was another pivotal domain. The high I-CVI obtained after clarification demonstrates expert agreement on the importance of early and appropriate nutritional support. Early ENT within 24–72 hours is associated with improved outcomes and reduced infectious complications.^5^ Exclusive PNT was assigned a higher risk score due to infection risks associated with central venous catheters.^26^

 Although several experts suggested the inclusion of additional biochemical markers (e.g., lactate, ferritin, procalcitonin), these were excluded to maintain feasibility and avoid dependence on non-routine laboratory data. CRP was retained as a pragmatic marker, reflecting inflammatory burden and its correlation with adverse nutritional trajectories.^27-30^ Elevated CRP has been associated with prolonged inflammation, poor nutritional tolerance, and extended hospital stays.^8^

 The seventh item, "risk-based nutritional interventions," reinforces the translational applicability of NutriRisKids-ICU. Rather than prescribing interventions, the tool provides guidance adaptable to institutional protocols, encouraging early, continuous nutritional monitoring, and multidisciplinary involvement to minimize malnutrition risk. 

 Although the sampling method used (snowball sampling) may present potential limitations related to selection bias, control measures were adopted to mitigate this risk, such as including professionals from different regions and specialties and collecting responses anonymously. This approach helped ensure diversity of experiences and greater independence of participants’ opinions. In addition, the inclusion of practical examples illustrating the application of NutriRisKids-ICU (Table 1) aims to facilitate understanding and future implementation of the tool in different clinical contexts, while preserving its feasibility and low operational cost. 

 Overall, the NutriRisKids-ICU differs from existing pediatric screening tools by focusing specifically on the critical care context, emphasizing rapid, low-cost, and clinically relevant variables. The high content validity index (CVI ≥0.90) obtained through expert consensus underscores its methodological robustness and potential clinical utility. 

 Among the strengths of this study are the multidisciplinary composition of the expert panel, the systematic Delphi approach, and the high agreement achieved across two validation rounds. Limitations include the intentional sampling of experts and the absence of clinical validation in patient populations. Future studies are needed to evaluate the tool’s sensitivity, specificity, and predictive accuracy in diverse PICU settings. 

 In conclusion, the NutriRisKids-ICU, a nutritional risk screening tool specifically developed for critically ill pediatric patients, demonstrated excellent content validity and was recognized by experts as a feasible, objective, and clinically relevant instrument for use in the PICU. Developed through expert consensus, the tool integrates theoretical evidence and clinical expertise, combining practicality with scientific rigor. Its use may enable earlier and more targeted nutritional interventions, supporting multidisciplinary decision-making and contributing to improved clinical outcomes. Nevertheless, further studies are required to evaluate its applicability, sensitivity, and specificity in different PICU settings. 

## Data Availability

The database that originated the article is available with the corresponding author.
